# Clustering of spontaneous recurrent seizures separated by long seizure-free periods: An extended video-EEG monitoring study of a pilocarpine mouse model

**DOI:** 10.1371/journal.pone.0194552

**Published:** 2018-03-20

**Authors:** Jung-Ah Lim, Jangsup Moon, Tae-Joon Kim, Jin-Sun Jun, Byeongsu Park, Jung-Ick Byun, Jun-Sang Sunwoo, Kyung-Il Park, Soon-Tae Lee, Keun-Hwa Jung, Ki-Young Jung, Manho Kim, Daejong Jeon, Kon Chu, Sang Kun Lee

**Affiliations:** 1 Department of Neurology, Comprehensive Epilepsy Center, Laboratory for Neurotherapeutics, Biomedical Research Institute, Seoul National University Hospital, Seoul, South Korea; 2 Department of Neurology, Gangnam Sacred Heart Hospital, Hallym University College of Medicine, Seoul, South Korea; 3 Department of Neurology, Ulsan University Hospital, Ulsan University College of Medicine, Ulsan, Korea; 4 Department of Neurology, Kyung Hee University Hospital at Gangdong, Seoul, South Korea; 5 Department of Neurology, Soonchunhyang University School of Medicine, Seoul, South Korea; 6 Department of Neurology, Seoul National University Hospital Healthcare System Gangnam Center, Seoul, South Korea; 7 Advanced Neural Technologies, Co., Seoul, South Korea; University of Modena and Reggio Emilia, ITALY

## Abstract

Seizure clustering is a common and significant phenomenon in patients with epilepsy. The clustering of spontaneous recurrent seizures (SRSs) in animal models of epilepsy, including mouse pilocarpine models, has been reported. However, most studies have analyzed seizures for a short duration after the induction of status epilepticus (SE). In this study, we investigated the detailed characteristics of seizure clustering in the chronic stage of a mouse pilocarpine-induced epilepsy model for an extended duration by continuous 24/7 video-EEG monitoring. A seizure cluster was defined as the occurrence of one or more seizures per day for at least three consecutive days and at least five seizures during the cluster period. We analyzed the cluster duration, seizure-free period, cluster interval, and numbers of seizures within and outside the seizure clusters. The video-EEG monitoring began 84.5±33.7 days after the induction of SE and continued for 53.7±20.4 days. Every mouse displayed seizure clusters, and 97.0% of the seizures occurred within a cluster period. The seizure clusters were followed by long seizure-free periods of 16.3±6.8 days, showing a cyclic pattern. The SRSs also occurred in a grouped pattern within a day. We demonstrate that almost all seizures occur in clusters with a cyclic pattern in the chronic stage of a mouse pilocarpine-induced epilepsy model. The seizure-free periods between clusters were long. These findings should be considered when performing *in vivo* studies using this animal model. Furthermore, this model might be appropriate for studying the unrevealed mechanism of ictogenesis.

## Introduction

A seizure cluster refers to a series of seizures that are closely grouped with shorter-than-average interictal periods [1]. Seizure clustering is commonly observed in patients with epilepsy, although the reported prevalence ranges widely from 7% to 83% [1–4]. Understanding the characteristics of seizure clusters is important not only because of their effects on the morbidity and mortality of patients with epilepsy [1] but also because such an understanding might help elucidate the mechanisms of seizure initiation and termination [1].

Seizure clustering has also been reported in several animal models of epilepsy: the rat and mouse pilocarpine models [5–9], the rat kainate model [10], and the rat model of perinatal hypoxia-ischemia [11]. The pilocarpine model is one of the most widely used models of temporal lobe epilepsy (TLE) [12–15], which is characterized by spontaneous recurrent seizures (SRSs) that occur following a latency period after pilocarpine-induced status epilepticus (SE). SRSs mimic the spontaneous seizures observed in patients with TLE [13–15]. Clustering of SRSs in both rat and mouse pilocarpine models has been described in few reports [5–7]. Mazzuferi *et al*. performed continuous 7-week electroencephalography (EEG) monitoring after SE induction in a mouse pilocarpine model and found that SRSs occurred in a particular pattern of clusters [8]. A recent study [9] demonstrated a circadian clustering of SRSs in the mouse pilocarpine model by performing continuous 4-week EEG monitoring after SE. However, detailed studies of the characteristics of seizure clusters in the chronic stage of the mouse pilocarpine model, beyond 7 weeks after SE, have not been conducted. Based on the recent concept of epileptogenesis, represented by the early onset and ongoing progression of the epilepsy process [16], and considering that most TLE patients suffer from spontaneous seizures over several years, understanding the characteristics of SRSs in the chronic stage of the mouse pilocarpine model is important. In this study, we investigated the characteristics of seizure clustering in the chronic stage of a mouse pilocarpine model for an extended duration.

## Materials and methods

### Generation of the experimental epilepsy model

A total of 118 five-week-old male C57BL/6 mice (18–22 g) were used for the generation of chronic epilepsy models. A single systemic injection of pilocarpine (400 mg/kg, intraperitoneal, Sigma, St. Louis, MO, USA) was given to each mouse for the induction of SE, as described previously [17–19]. To reduce peripheral muscarinic effects, methylscopolamine (1 mg/kg, intraperitoneal, Sigma, St. Louis, MO, USA) was administered to the mice 30 min before the pilocarpine injection. To interrupt the prolonged seizure, diazepam (5 mg/kg, intraperitoneal) was injected at 40 min after the onset of SE. SE was defined as continuous tonic-clonic seizures following several discontinuous convulsive seizures (stage ≥ 4) [20].

### Electrode implantation and video-EEG monitoring

**Fig 1:**
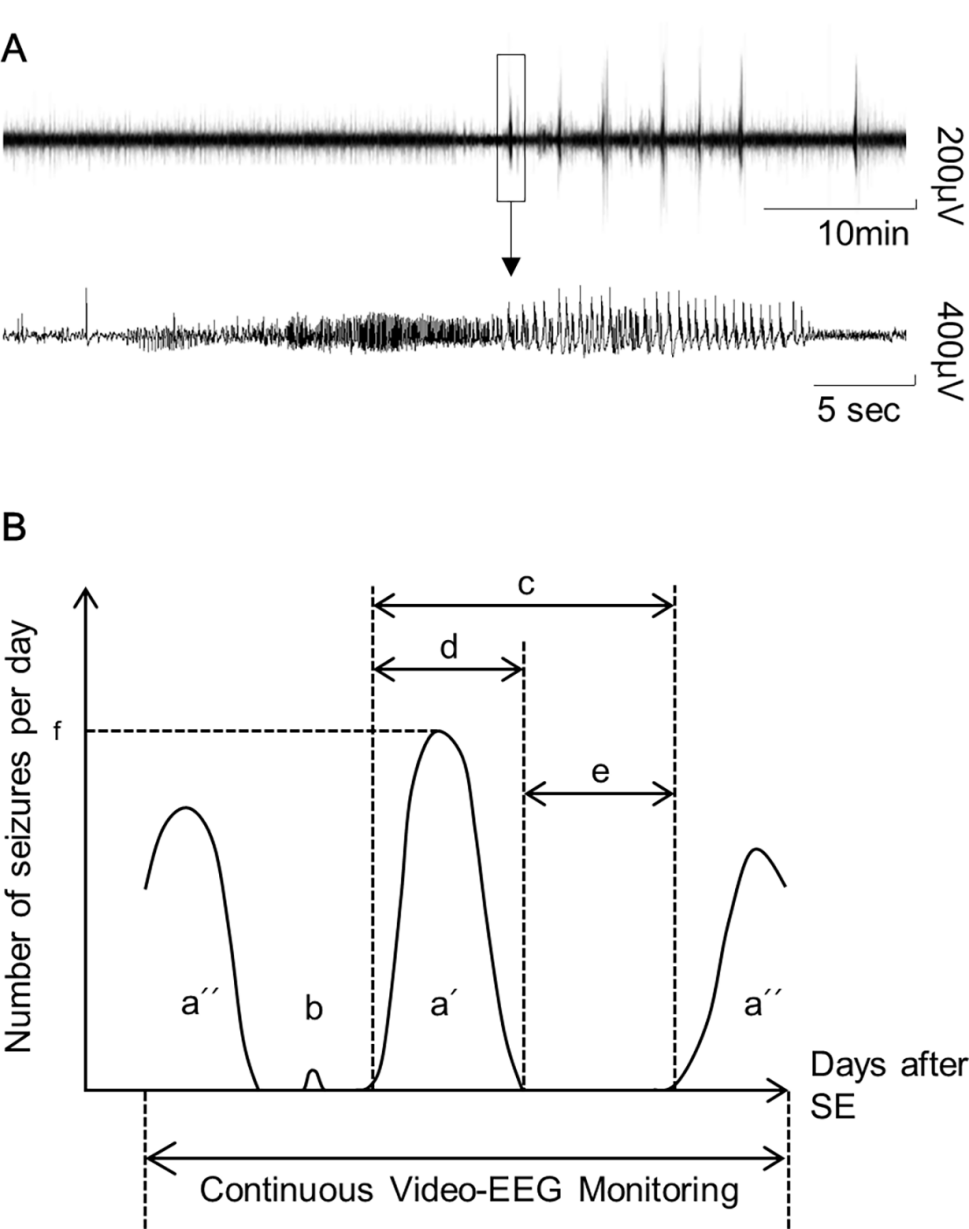
**Typical EEG pattern of spontaneous seizures (A) and schematic description of the parameters (B).** (A) Typical EEG recording of seizure clusters (upper) and a spontaneous seizure (lower). Upper: The 2-hour EEG trace shows that seizures occurred in a cluster in the latter part of the trace. A seizure in the upper EEG trace (black box) is presented in the lower EEG trace. (B) a (both a’ and a”): seizure cluster, a’: fully monitored (FM) cluster, a”: seizure cluster that was not fully monitored, b: seizure outside a seizure cluster, c: cluster interval, d: cluster duration, e: seizure-free period, f: peak seizure frequency.

For EEG surgery and recording, long-term video-EEG monitoring was performed as described previously [17,19]. Electrode implantation surgery was performed more than 7 days before the initiation of recording to provide sufficient time for recovery from the surgery. To anesthetize the animals, ketamine (90 mg/kg) and xylazine hydrochloride (40 mg/kg) were injected intraperitoneally. Surgery was performed using a stereotaxic apparatus (Kopf Instruments). Recordings were obtained using skull screws (stainless steel, 1.0 mm in diameter), which were positioned in–1.8 mm AP and –2.1 mm ML from bregma with grounding over the cerebellum. Local anesthetic (lidocaine 1%, transdermal) was used for perioperative analgesia. Electrical activities were recorded after amplification (×1200), bandpass filtering at 0.1–70 Hz, and digitization with a 400-Hz sampling rate (AS 40) using a digital system (Comet XL, Astro-Med, Warwick, RI, USA). Video-EEG signals were continuously recorded for 24 hours per day, and seizure activities were analyzed offline using PSG Twin 4.3 software (Astro-Med, West Warwick, RI, USA). EEG recordings were reviewed by visual inspection. An electrographic seizure was defined as a discharge showing a change in EEG amplitude (>2×baseline) and frequency (repetitive spiking with a frequency of 4-12/s) that lasted for a minimum of 10 s (Fig 1A [lower]). Behavioral seizures were confirmed via video monitoring according to the Racine staging system [20]. SRS was defined as a convulsive seizure (stage ≥ 4) detected on EEG.

The mice were sacrificed via cervical dislocation after completion of video-EEG monitoring. All of the procedures involving animal care and handling were approved by the Institutional Animal Care and Use Committee at Seoul National University Hospital, and all of the experiments were performed in accordance with relevant guidelines and regulations.

### Analysis of seizure clusters and definition of cluster parameters

To analyze the seizure clusters, the numbers of SRSs per day were counted. A seizure cluster was defined as the occurrence of one or more seizures per day for at least three consecutive days and at least five seizures within the cluster period (Fig 1B [a]). When a seizure cluster was fully monitored from the onset to the end of the cluster, the cluster was categorized as a fully monitored cluster (FM cluster, Fig 1B [a’]); in some mice, the EEG monitoring was started or ended during a seizure cluster period (Fig 1B [a”]). The cluster interval was defined as the time (in days) between the onset of seizure clusters (Fig 1B [c]). The cluster duration was defined as the time (in days) from the onset to the end of the seizure cluster (Fig 1B [d]). The period (in days) between seizure clusters without any other seizures was defined as the seizure-free period (Fig 1B [e]). Peak seizure frequency (Fig 1B [f]) was defined as the peak number of SRSs per day within the FM cluster.

As it was technically challenging to perform video-EEG monitoring for a particularly long duration in each animal due to electrode detachment or electric wire damage, we monitored the animals in different periods of the chronic stages. We classified the animals into two different groups (chronic phase 1 and chronic phase 2) according to their monitoring periods. Chronic phase 1 was defined as the period from 6 to 14 weeks after SE, and chronic phase 2 was defined as the period beginning 15 weeks after SE.

#### Statistical analysis

Significant differences between two groups were assessed using Student’s t test. The paired t test was used to evaluate differences in paired data. Pearson’s correlation coefficient was used to measure the correlation between two variables. SPSS 21 software was used for all statistical analyses, and a two-tailed *p-*value less than 0.05 was considered significant.

## Results

Among the 106 mice in which pilocarpine was injected, 49 (46.2%) mice died during the course of pilocarpine-induced SE, and 16 (15.1%) mice did not develop SE. EEG recordings were performed in the remaining 41 mice, but they were discontinued in 14 mice due to dislodged or noisy electrodes during recording. Consequently, 27 mice with SE and subsequent SRSs were analyzed.

**Fig 2:**
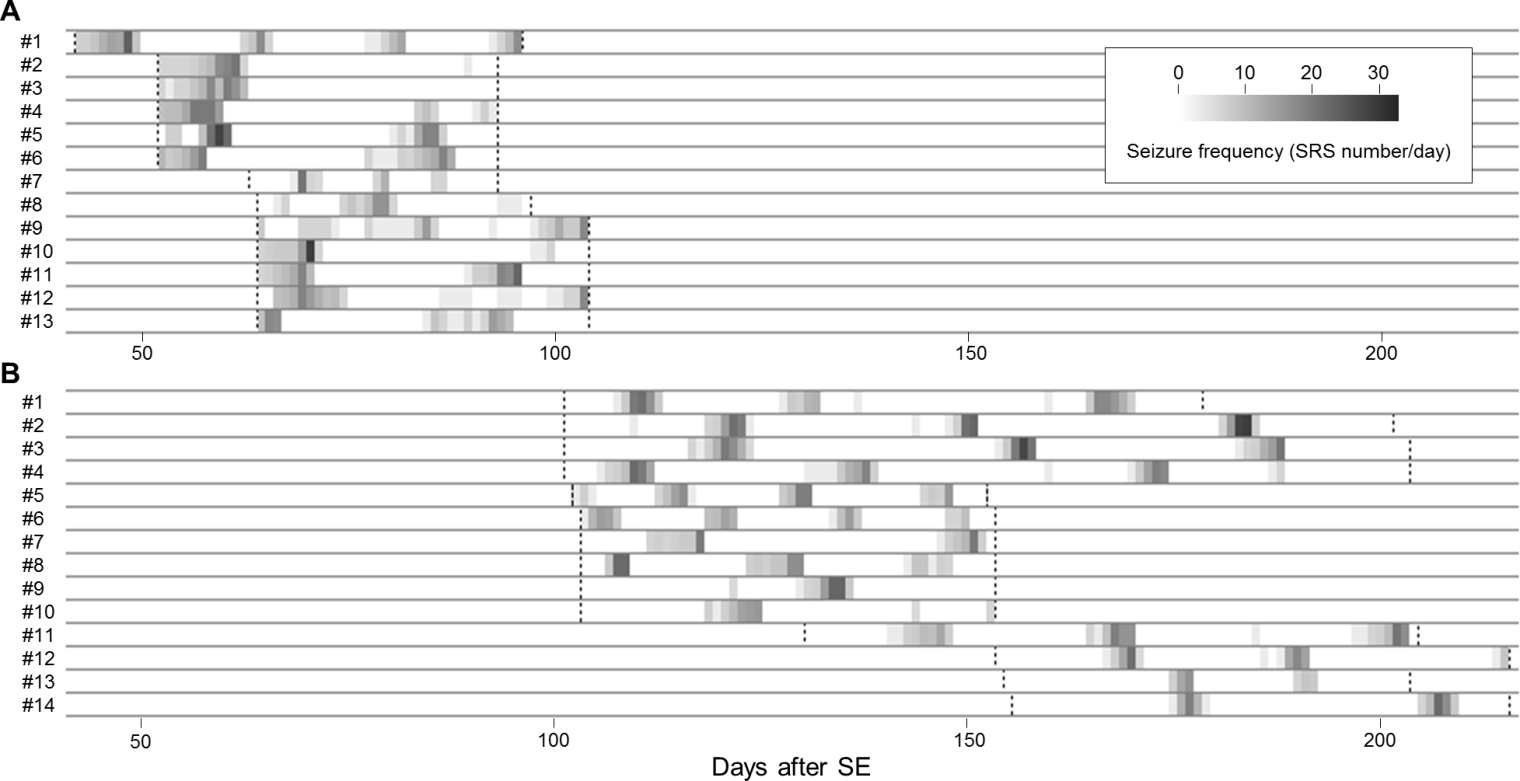
Daily frequency of spontaneous recurrent seizures in all individual mice. The seizure frequencies (spontaneous recurrent seizures/day) in individual mice are represented by the gray bar: light color indicates a low seizure frequency, and dark color indicates a high seizure frequency. The start and end of the monitoring period are marked by the vertical dotted lines. (A) Chronic phase 1. (B) Chronic phase 2. SE = status epilepticus, SRS = spontaneous recurrent seizure.

**Fig 3:**
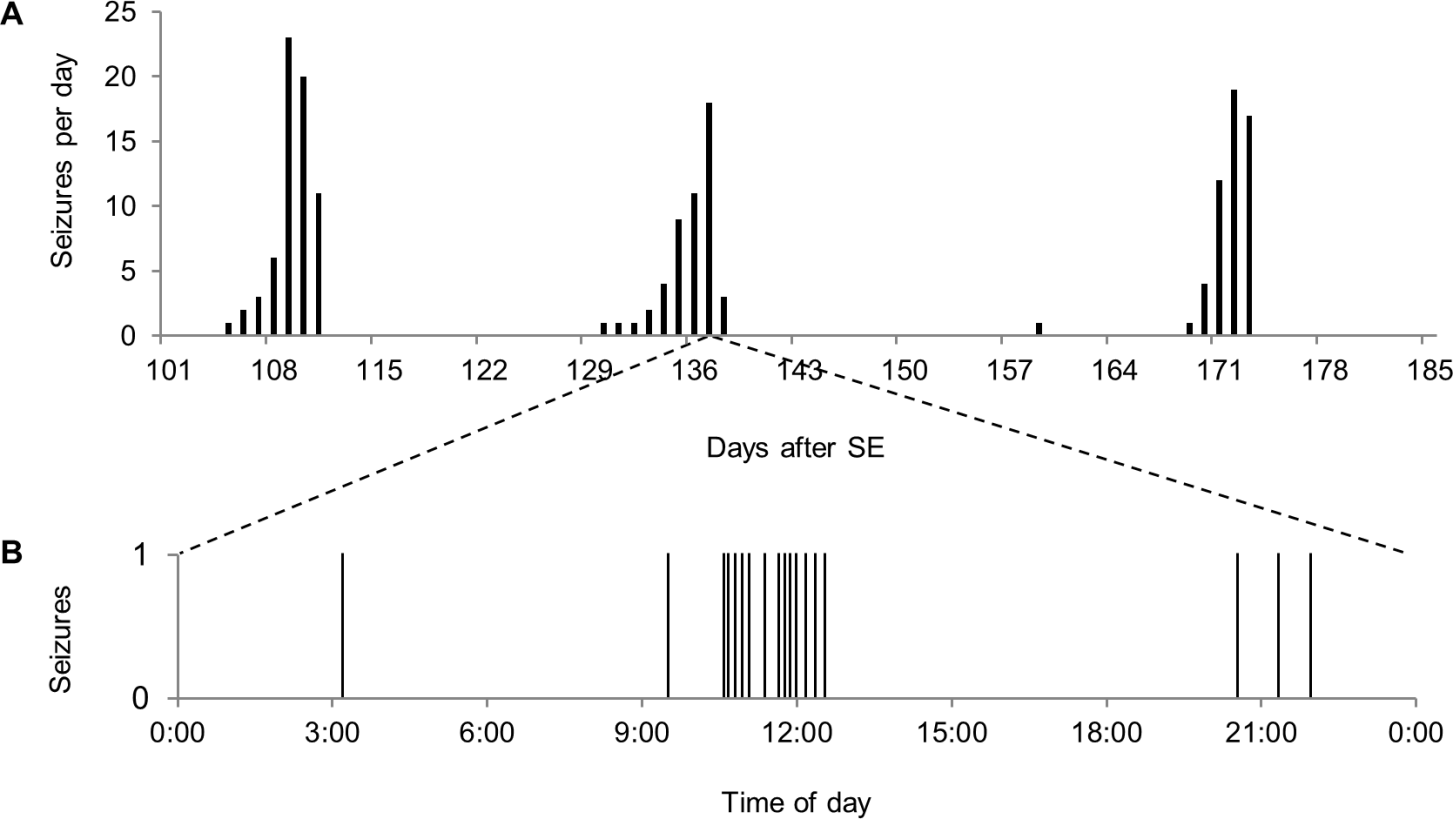
Video-EEG monitoring data from a representative animal. Video-EEG monitoring data from a representative animal. (A) The number of spontaneous seizures per day. (B) Raster plot of the seizure frequency on the day of peak seizure frequency. The data are extracted from animal #4 in the chronic phase 2 group.

Continuous video-EEG recording was performed for 53.7±20.4 days (mean±SD). All of the mice displayed seizure clusters, as shown in Fig 2. Among the observed seizures, 97.0% occurred within a cluster period. A total of 62 seizure clusters were observed, with a mean of 2.3±1.0 seizure clusters per mouse. The seizure clusters showed unimodal curves and were followed by seizure-free periods, which occurred in a cyclic pattern, as shown in Fig 2 and Fig 3A. The mean seizure-free period was 16.3±6.8 days. Moreover, when a mouse experienced multiple seizures in a day, the seizures were not evenly distributed, as shown in Fig 3B.

Among the 62 seizure clusters, 47 clusters were fully monitored. The mean duration of FM clusters was 5.7±2.0 days, and the mean cluster interval was 22.7±8.7 days. The mean seizure frequency during the FM clusters was 8.0±3.8 per day, and the mean peak seizure frequency was 17.3±6.5 per day.

**Fig 4:**
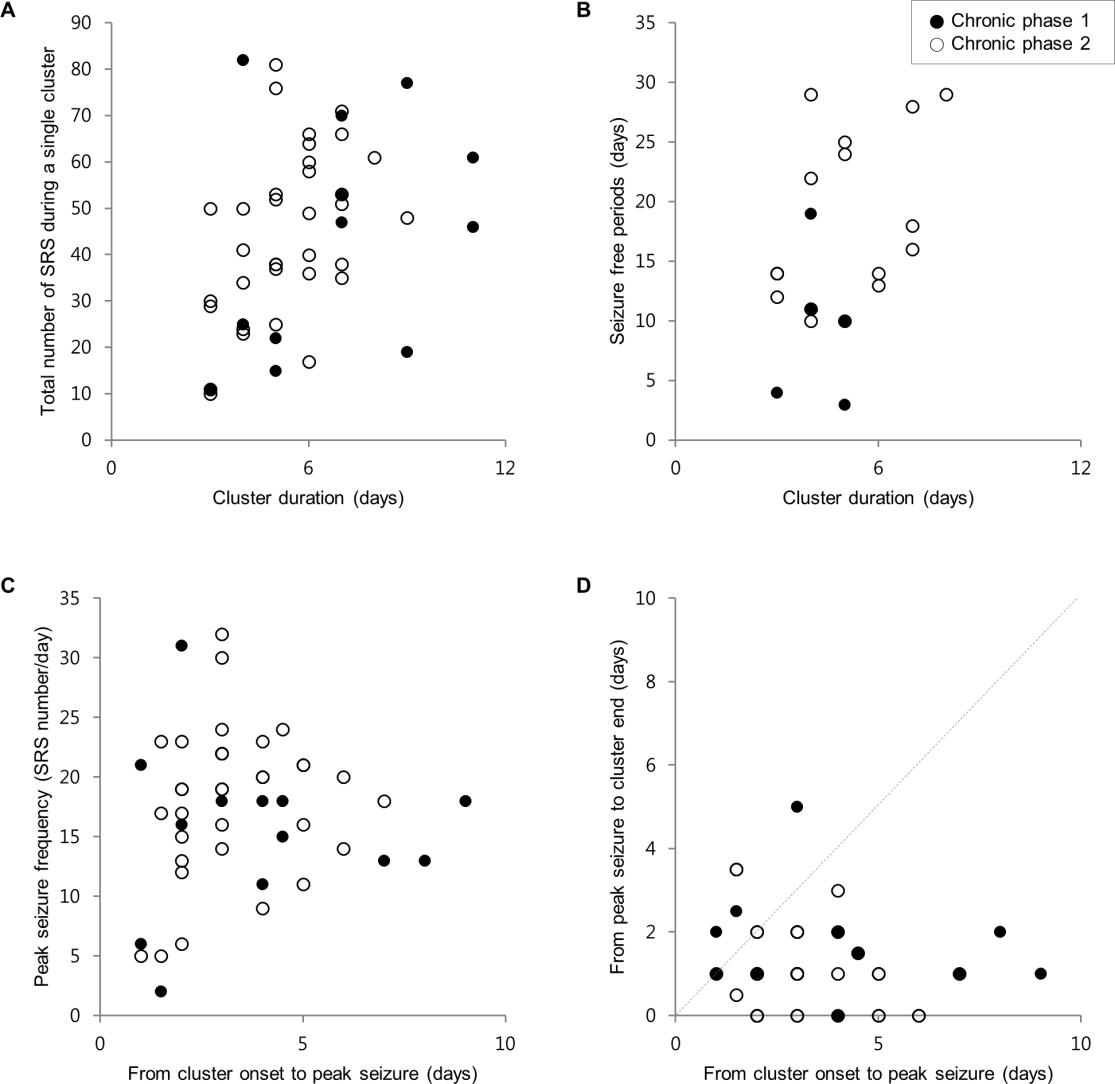
Scatter plots of various parameters. Scatter plots of cluster duration vs total number of spontaneous recurrent seizures during a single cluster (A), cluster duration vs duration of the following seizure-free period (B), number of days from cluster onset to the day of peak seizure frequency vs peak seizure frequency (C), and number of days from cluster onset to the day of peak seizure frequency vs number of days from the day of peak seizure frequency to cluster end (D). The dotted line represents a slope of 1.

The parameters of the seizure clusters were highly variable among the clusters (Fig 4) and within each mouse. The cluster duration ranged from 3 to 11 days. During a single cluster, the total number of SRSs ranged from 10 to 82. The cluster duration was correlated with the total number of SRSs within the cluster (*r* = 0.4, *p* = 0.006, Fig 4A). The duration of seizure-free periods following seizure clusters ranged from 4 to 29 days and was not correlated with the duration of the preceding clusters (Fig 4B). In addition, the peak seizure frequency of the cluster did not correlate with the number of days from cluster onset to the day of peak seizure frequency (Fig 4C). The seizure clusters presented as unimodal curves, but the day of peak seizure frequency was not in the middle of the cluster. The seizure frequency gradually increased over time, and after the peak, the seizure cluster rapidly ended (Fig 4D). The number of days from cluster onset to the day of peak seizure frequency was greater than the number of days from the day of peak seizure frequency to cluster end (3.4±1.9 vs 1.2±1.1, *p*<0.0001, Table 1).

**Table 1:** Characteristics of spontaneous recurrent seizures in a mouse pilocarpine-induced epilepsy model in chronic phase.

	Total	Chronic phase 1	Chronic phase 2	*p*-value*
	(n = 27)	(n = 13)	(n = 14)	
Duration from SE to monitoring onset (days)	84.5± 33.7 (42–155)	57.6±7.5 (42–64)	115.2±22.3 (101–155)	<0.0001
Duration from SE to monitoring end (days)	140.2±46.8 (92–214)	96.8±5.3 (92–103)	180.5±27.0 (151–214)	<0.0001
Duration of monitoring (days)	53.7±20.4 (30–102)	40.2±5.4 (30–54)	66.3±21.2 (49–102)	<0.0001
Total number of SRSs during the monitoring period	105.0±41.8 (40–193)	96.5±32.2 (40–146)	112.8±49.0 (40–193)	0.320
Seizures occurring within clusters (%)	97.0±7.4 (62.5–100)	94.6±10.2(62.5–100)	99.0±1.5 (94.6–100)	0.144
Total number of SRSs within a single cluster†	44.0±19.8 (10–82)	42.5±24.7 (11–82)	44.6±18.0 (10–81)	0.758
Seizure frequency over the total recording period (number of SRSs/day)	2.0±0.6 (1.1–3.4)	2.4±0.6 (1.3–3.4)	1.7±0.3 (1.1–2.1)	0.004
Seizure frequency within clusters† (number of SRSs/day)	8.0±3.8 (2.1–20.5)	6.8±4.7 (2.1–20.5)	8.7±3.4 (5.4–16.7)	0.181
Peak seizure frequency† (number of SRSs/day)	17.3±6.5 (2–32)	15.4±7.1 (2–31)	18.0±6.3 (5–32)	0.228
Cluster duration† (days)	5.7±2.0 (3–11)	6.6±2.7 (3–11)	5.4±1.6 (3–9)	0.135
Duration from cluster onset to the day of peak seizure frequency† (days)	3.4±1.9 (1–9)	4.0±2.6 (1–9)	3.3±1.5 (1–7)	0.258
Duration from the day of peak seizure frequency to cluster end† (days)	1.2±1.1 (0–5)	1.7±1.2 (0–5)	1.1±1.0 (0–3.5)	0.107
Cluster interval (days)	22.7±8.7 (7–37)	17.2±9.0 (7–33)	24.6±8.0 (14–37)	0.054
Inter-cluster seizure-free period (days)	16.3±6.8 (4–29)	14.8±6.0 (4–23)	17.0±7.3 (7–29)	0.436

Data are reported as the means ± SD (range).

*Comparison between the chronic phase 1 and chronic phase 2 groups.

†Data from fully monitored clusters only. Abbreviations: SE = status epilepticus, SRS = spontaneous recurrent seizure

Thirteen mice in the chronic phase 1 group started to undergo video-EEG monitoring at 57.6±7.5 days after SE, and monitoring was continued for 40.2±5.4 days. Fourteen mice in the chronic phase 2 group started to undergo monitoring at 115.2±22.3 days after SE, and this monitoring was continued for 66.3±21.2 days. Most of the characteristics of the seizure clusters were similar between the groups (Table 1). The chronic phase 1 group had more frequent seizures over the total monitoring period, with 2.4±0.6 SRSs/day in the chronic phase 1 group and 1.7±0.3 SRSs/day in the chronic phase 2 group. The cluster intervals tended to be longer in the chronic phase 2 group, with 17.2±9.0 days in the chronic phase 1 group and 24.6±8.0 days in the chronic phase 2 group (*p* = 0.054).

We applied our definition of a seizure cluster to the data from a previous study that was performed in the earlier period of a mouse pilocarpine model, from the day of SE to 49 days after SE [8] (Table 2). During that period, 77.8% of the seizures occurred within a cluster period. The cluster interval was 1 to 18 days, and the inter-cluster seizure-free period was 1 to 12 days. The peak seizure frequency was 2 to 24.

**Table 2:** Comparison of characteristics of spontaneous recurrent seizures with a previous study using our definition of seizure cluster.

	Early epileptogenic phase, (Data from Mazzuferi *et al*[8]†, n = 7)	Chronic phase (Our data, n = 27)	*p*-value
Duration from SE to monitoring onset (days)	0	84.5± 33.7 (42–155)	
Duration from SE to monitoring end (days)	49	140.2±46.8 (92–214)	
Duration of monitoring (days)	49	53.7±20.4 (30–102)	
Total number of SRSs during the monitoring period	71.3±52.3 (26–181)	105.0±41.8 (40–193)	0.917
Seizures occurring within clusters (%)	77.8±20.2 (52.8–100)	97.0±7.4 (62.5–100)	<0.0001
Total number of SRSs within a single cluster*	19.8±16.7 (5–66)	44.0±19.8 (10–82)	0.135
Seizure frequency over the total recording period (number of SRSs/day)	1.4±1.1 (0.5–3.7)	2.0±0.6 (1.1–3.4)	0.246
Seizure frequency within clusters† (number of SRSs/day)	4.4±3.3 (1.3–14.3)	8.0±3.8 (2.1–20.5)	0.380
Peak seizure frequency* (number of SRSs/day)	7.5±5.4 (2–24)	17.3±6.5 (2–32)	0.331
Cluster duration* (days)	4.5±1.7 (2–9)	5.7±2.0 (3–11)	0.562
Cluster interval (days)	5.9±4.4 (1–18)	22.7±8.7 (7–37)	0.001
Inter-cluster seizure-free period (days)	4.6±2.6 (1–9)	16.3±6.8 (4–29)	0.008

Data are reported as the means ± SD (range).

*Data from fully monitored clusters only. Abbreviations: SE = status epilepticus, SRS = spontaneous recurrent seizure.

† Our definition of seizure cluster was applied to the data which were obtained from the figure 8 of the article.

## Discussion

In this study, we investigated the distribution of SRSs in a mouse pilocarpine model for an extended period during the chronic stage. Nearly all SRSs occurred within clusters, and these clusters were followed by long seizure-free periods. Although the parameters of clusters were variable among the mice and among the clusters, each mouse consistently demonstrated a cyclic pattern of seizure clusters in both chronic phase 1 and chronic phase 2. Moreover, the seizures were clustered even within a single day.

All of the mice demonstrated SRS clustering, and most of the seizures occurred within clusters, which were followed by long seizure-free periods. The mean cluster interval and mean seizure-free period were longer than we initially expected. In addition, less than 3% of the seizures occurred outside a cluster. When our definition of a seizure cluster was applied to the data from a previous study that was performed in the earlier period (from the day of SE to 49 days after SE) of a mouse pilocarpine model [8], the characteristics of the seizure clusters were slightly different from the characteristics observed in the present study. All of the mice showed seizure clusters similar to the mice in the present study, but a smaller percentage of seizures occurred within a cluster period. The cluster interval and the inter-cluster seizure-free period were shorter than those of the present study. In addition, the peak seizure frequency was lower than that of our study. Based on these differences in results between studies, we infer that seizure clustering becomes more evident with time after SE.

The seizures occurred in a grouped pattern, even within a day. A recent study reported the circadian clustering of SRSs in a mouse pilocarpine model [9]. In that study, the power frequency of EEG was analyzed along with the movement/activity of the animals at an early stage of epileptogenesis, from SE induction to 28 days after SE. The authors revealed the striking clustering of SRSs at the transition between the sleep and activity stages. Since EEG power frequency analysis was not performed in the present study and the light/dark cycle was not tightly controlled, we did not analyze the circadian pattern of seizure clustering in relation to the sleep cycle. However, uneven distributions of seizures within a day were observed in both our study and another recent study [9].

The clustering and cyclicity of SRSs in rat pilocarpine models have been described before [5–7]. Arida *et al*. monitored epileptic rats for an extended period at the chronic stage, until 135 days after the first SRS [5]. However, the seizures were monitored only visually without EEG, and the duration of seizure clusters was not assessed. Compared with the data obtained from rat models [5–7], the durations of seizure clusters and seizure-free periods are longer in the mouse model studied here. However, further studies are required to verify the generalizability of this observation. In addition, the durations of the seizure-free periods were highly variable within a model for both the mouse and rat epilepsy models [5].

Most of the parameters of the seizure clusters were similar between the chronic phase 1 and chronic phase 2 groups. Only the seizure frequency over the total monitoring period differed between the two groups. This difference is likely due to the inclusion of more seizure-free periods during the monitoring of the mice in the chronic phase 2 group. In addition, it is interesting that the cluster intervals tended to be longer in the chronic phase 2 group, although the intervals were not significantly different from those of the phase 1 group. Moreover, the cluster intervals in our study were longer than those observed in early epileptogenic periods, as mentioned above; [8,9] therefore, it is possible that the cluster interval increases with time. This possibility should be investigated in the future by performing prolonged EEG monitoring from the early epileptogenic period to the late chronic stage in individual mice.

Considering the long durations of the seizure-free periods and cluster intervals in the mouse pilocarpine model, we recommend that *in vivo* studies using this model perform long-term EEG monitoring, i.e., for at least several weeks. Mouse pilocarpine models have been widely used in drug development studies; however, many studies have not performed long-term monitoring, with some studies monitoring for only certain periods of a day [21–23]. With insufficient monitoring periods, it would be difficult to distinguish the effect of a drug candidate from the effect of the seizure cycle *per se*. In addition, animals should be monitored 24 hours a day because seizures can occur in a clustered pattern, even within a day.

The fundamental mechanism underlying seizure clusters remains unclear. A previous study [24] demonstrated that pharmaco-resistant seizures occur in clusters and suggested that the seizures have an inherent capacity to trigger seizures, i.e., that they are ‘self-triggering’. Based on that study, Ferastraoaru *et al*. hypothesized that seizures occur in clusters due to a failure of inhibitory mechanisms and demonstrated that intracluster seizures are shorter than the last seizure in a cluster or isolated seizures [4], supporting their hypothesis. The mouse pilocarpine-induced epilepsy model could be used to illuminate the mechanism of seizure clustering and ictogenesis by comparing the histopathologic or molecular characteristics of animals within a cluster period to those in the seizure-free period.

One of the limitations of our study is the use of cortical electrodes and the limitation of monitoring to seizures with a Racine stage of 4 or higher. However, previous studies [8,9] have demonstrated that seizures become more severe over time after the induction of SE, and almost all of the SRSs in the chronic stage were stage 4–5. Therefore, we assume that we did not fail to detect many mild seizures that could have been detected using depth electrodes.

## Conclusions

This is the first study to demonstrate the clustering of SRSs during the extended chronic phase in a mouse pilocarpine-induced epilepsy model. Our findings reaffirm that clustering of SRSs continues over time. Moreover, we demonstrate that the seizure-free periods between clusters are substantially long and that seizures occur in a grouped pattern, even within a day, which supports the necessity of long-term continuous 24/7 EEG monitoring. These findings are highly informative for those planning to design future *in vivo* studies with mouse pilocarpine-induced epilepsy models. Although the clustering and cyclic characteristics of SRSs complicate the design of *in vivo* studies using this model, these characteristics might be valuable for studying the mechanisms of seizure clustering and ictogenesis.
