# Correction: Clustering of spontaneous recurrent seizures separated by long seizure-free periods: An extended video-EEG monitoring study of a pilocarpine mouse model

**DOI:** 10.1371/journal.pone.0240544

**Published:** 2020-10-06

**Authors:** Jung-Ah Lim, Jangsup Moon, Tae-Joon Kim, Jin-Sun Jun, Byeongsu Park, Jung-Ick Byun, Jun-Sang Sunwoo, Kyung-Il Park, Soon-Tae Lee, Keun-Hwa Jung, Ki-Young Jung, Manho Kim, Daejong Jeon, Kon Chu, Sang Kun Lee

The Data Availability statement for this paper [1] is incorrect. The correct statement is: Quantitative data underlying results reported in Fig 4 and Tables 1 and 2 are provided in S1 Table. The individual-level EEG data are available upon request to the corresponding author.

The raw data underlying Fig 4 and Tables 1 and 2 are missing from the list of Supporting Information. The authors have provided the data as Supporting Information file S1 Table. With this correction, all relevant data are now provided.

The authors have also provided additional methodological information about the pilocarpine-induced epilepsy model and measures that were taken during the study in consideration of animal welfare here:

The mortality rates for murine pilocarpine-induced epilepsy models vary depending on the experimental protocols (25–100%). [2, 3] The mortality rate in this model is affected by many factors including the mouse strain, pilocarpine dose, status epilepticus (SE) duration, and pre/post treatments of pilocarpine induction. It was previously reported that when pilocarpine-induced epilepsy model is generated in mouse, only ~60–70% of mice develop SE; of the mice which develop SE, approximately half die (about 30–40% of the initial sample) and the remaining half (about 30–40%) can be used as the mouse model of epilepsy. [2]
For the current study [1], the authors informed the Institutional Animal Care and Use Committee (IACUC) at Seoul National University Hospital that there was an expected mortality rate of 40–60%, and the IACUC approved a sample size of 160 animals. As was reported in the article [1], “49 (46.2%) mice died during the course of pilocarpine-induced SE”. In more detail, 19 mice died immediately after injection of pilocarpine, 27 mice survived after pilocarpine induced SE but died within two weeks, and 3 mice died during electrode implantation surgery. Excluding the mice that died during the electrode surgery, 46 mice (43.4%) died during the generation of epilepsy mouse model.
During the study, animals were maintained in a facility accredited by AAALAC International (#001169), in accordance with Guide for the Care and Use of Laboratory Animals 8th edition, NRC (2010). Skilled personnel checked health of the animals daily. Especially after induction of SE, animals were monitored closely for three days. For animals with poor recovery, intraperitoneal 0.2% glucose or oral water was administered twice a day. Humane endpoint criteria were applied in accordance with the Humane Endpoints in Animal Experiments for Biomedical Research. Specifically, animals were to be euthanized if they exhibited the following: 20% body weight loss for 7 days or inability to eat or drink (less than 40% of the previous 3 days) with hunched posture, unsmooth coat, and labored breathing. 49 mice died during the course of the experiments. However, none of these mice fulfilled the humane endpoint criteria for euthanasia; therefore, they were not euthanized.

## Supporting information

S1 Table Individual level quantitative data. Daily frequency of spontaneous recurrent seizures in individual mice. The number of seizures observed during continuous video-EEG monitoring is displayed in the table. These data support the results of Fig 4, Table 1, and Table 2.
(XLSX)
